# Older mothers and increased impact of prenatal screening: stable livebirth prevalence of trisomy 21 in the Netherlands for the period 2000–2013

**DOI:** 10.1038/s41431-017-0075-1

**Published:** 2018-01-12

**Authors:** Maurike D. de Groot- van der Mooren, Saskia Tamminga, Dick Oepkes, Michel E. Weijerman, Martina C. Cornel

**Affiliations:** 1 Department of Pediatrics, VU University Medical Center, Amsterdam, The Netherlands; 2 Department of Clinical Genetics, VU University Medical Center, Amsterdam, The Netherlands; 3 Amsterdam Public Health research institute, VU University Medical Center, Amsterdam, The Netherlands; 4 Dutch Working group Prenatal Diagnosis and Therapy, Leiden, The Netherlands; 5 Department of Pediatrics, Alrijne hospital, Leiderdorp, The Netherlands

## Abstract

In the Netherlands, there is no registry system regarding the livebirth prevalence of trisomy 21 (T21). In 2007, a national screening programme was introduced for all pregnant women, which may have changed the livebirth prevalence of T21. The aim of this study is to analyse trends in factors that influence livebirth prevalence of T21 and to estimate the livebirth prevalence of T21 for the period of 2000–2013. National data sets were used on the following: (1) livebirths according to maternal age and (2) prenatal testing and termination of pregnancy (ToP) following diagnosis of T21. These data are combined in a model that uses maternal age-specific risk on T21 and correction factors for natural foetal loss to assess livebirth prevalence of T21. The proportion of mothers aged ≥ 36 years has increased from 12.2% in 2000 to 16.6% in 2009, to gradually decrease afterwards to 15.2% in 2013. The number of invasive tests performed adjusted for total livebirths decreased (5.9% in 2000 vs. 3.2% in 2013) with 0.18% a year (95% CI: −0.21 to −0.15; *p*   < 0.001). Following invasive testing, a higher proportion of foetuses was diagnosed with T21 (1.6% in 2000 vs. 4.8% in 2013) with a significant increase of 0.22% a year (95% CI: 0.18–0.26; *p* < 0.001). The proportion of ToP subsequent to T21 diagnosis was on average 85.7%, with no clear time trend. This resulted in a stable T21 livebirth prevalence of 13.6 per 10,000 livebirths (regression coefficient −0.025 (95% CI: −0.126 to 0.77; *p* = 0.60).

## Introduction

Trisomy 21 (T21) or also called Down Syndrome (DS), is the most common chromosomal disorder among liveborn infants and is associated with intellectual disability and other serious morbidity [1]. Studies have shown that T21 livebirth prevalence has been influenced mainly by two trends with counteracting effects [2–7]. Firstly, in developed countries, a rise in maternal age increases the chance of giving birth to a child with T21 [8]. For the Northern Netherlands, the percentage of mothers aged > 35 years of age has also increased from 12 to 17 percent in the period 1993–2004 [5]. Secondly, more precise and advanced technologies for T21 screening during pregnancy have become available and are being offered to pregnant women, which may have changed T21 livebirth prevalence. In the Netherlands, before 2007, prenatal screening with the First-trimester Combined screening Test (FCT) was only recommended for women older than 35 years. However, women over 35 years could also opt for direct prenatal diagnosis through an amniocentesis (AC) or chorion villous sampling (CVS) [9]. A national program for prenatal screening started in 2007 consisting of all pregnant women receiving information about FCT in the first trimester and furthermore offering a structural anomaly scan in the second trimester [10]. Risk assessment for trisomy 21, 13, and 18 with the FCT is based on maternal age, foetal nuchal translucency thickness and concentrations of maternal serum-free β-human chorionic gonadotrophin and pregnancy-associated plasma protein-A. If a woman receives a “high-risk” FCT result ( ≥ 1:200) or abnormal ultrasound findings are present, prenatal diagnostic testing by CVS or AC is offered for follow-up. The purpose of prenatal DS screening is to enable autonomous informed decision making with regard to carrying pregnancy to term or termination of pregnancy (ToP) [11, 12]. Studies have shown that many women decline follow-up invasive testing, sometimes due to the risk of miscarriage of 0.11–0.22% [13–15]. Studies have shown that the vast majority (85–95%) of the women that receive a foetal T21 diagnose terminate their pregnancy [16]. It is unclear if actively informing all pregnant women about prenatal screening tests, starting around 2004, and resulting in a national screening program in 2007, led to a change in livebirth prevalence of T21. The aims of this study were to analyse trends in factors that influence T21 livebirth prevalence and to estimate livebirth prevalence of T21 in the Netherlands for the period 2000–2013.

## Subjects and methods

### Model

**Fig. 1:**
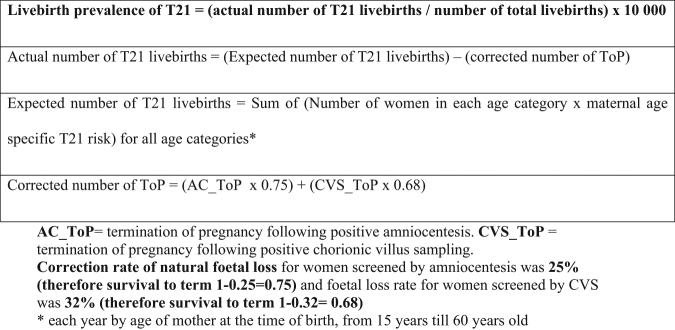
Model for estimating livebirth prevalence of T21. Correction rates of natural foetal loss for women screened by amniocentesis was 25% (therefore survival to term 1−0.25 = 0.75) and foetal loss rates for women screened by CVS were approximately 32% (therefore survival to term 1−0.32 = 0.68)* each year by age of mother at the time of birth, from 15 years till 60 years old. AC_ToP termination of pregnancy following positive amniocentesis, CVS_ToP termination of pregnancy following positive chorionic villus sampling

In this study, we will estimate T21 livebirth number in the Netherlands on the basis of a model with following variables: (1) number of total livebirths specified for maternal age; (2) maternal age-specific T21 risk; and (3) number of ToP in the case of T21. Livebirth prevalence is defined as the number of liveborn children with T21 per 10,000 livebirths (Fig. 1).

### Data sets

In order to assess the numbers of the above-mentioned variables and relevant trends of T21 in the Netherlands, the following two data sets were combined: the Central Bureau of Statistics (CBS) and the Working Party on Prenatal Diagnosis and Therapy (WPDT). The CBS collects and processes national data on a mandatory basis, anonymously. The published data covers multiple societal aspects of the Dutch population, including numbers and specifics with regard to childbirth. In this study, the total number of annual livebirths and maternal age distribution (each year by age of mother in completed years at the time of birth) were used. In the table of CBS, some children have been included in a specific year in which they were reported to the municipal administration, while they were born in the previous or next year. Therefore, numbers are slightly different than the CBS tables online [17]. The WPDT is part of the Dutch Society for Clinical Genetics (VKGN) and the Dutch Society for Obstetricians and Gynaecologists (NVOG). Since 1991, the WPDT collects the data in the Netherlands concerning prenatal T21 screening. The WPDT annual report contains statistics on performed prenatal diagnoses by AC or CVS, diagnosed T21 cases and the number of T21 pregnancies terminated after diagnosis. Different WPDT reports are circulating, we used the final versions [18]. This data set does not contain personal information and therefore is not privacy-sensitive. The Medical Ethical Committee of VU University Medical Center stated that no permission needed to be granted for this study in accordance with Dutch research legislation (WMO).

### Expected number of T21 livebirths

By multiplying the number of women that delivered a liveborn child in each age category by the age-specific T21 risks, and summing that for all maternal age categories, the annual number of expected T21 children could be estimated. The maternal age-specific risks of T21 proposed by Morris were used [19]. Compared to the other models, the analysis of Morris et al is the most recent model and based on the largest data set. Furthermore, data from Morris et al provide some evidence to show that the risk does not continue to increase exponentially for women over age 45 as previous estimates assume [20]. With the expected number of T21 livebirths, it was possible to estimate the “natural prevalence”. The natural prevalence is the number of children with T21 that would have been born in the absence of prenatal screening and selective abortion per 10,000 livebirths.

#### Actual number of T21 livebirths

Next, the expected number of T21 livebirths is corrected for the effect of prenatal testing and subsequent termination of pregnancies. In 2011 and 2013, the WPDT annual reports contained no information on the number of ToPs of the centre in Nijmegen. We have estimated this number by assuming that, in Nijmegen, the proportion of ToP (separately for AC and CVS) was similar to that in the other centres [21]. Still, the number of induced abortions does not result in an equivalent reduction in the number of T21 livebirths due to the risk of natural foetal loss. Many of these induced abortions would not have survived to term and would not have been diagnosed, as miscarriages are generally not karyotyped. The risk of natural foetal loss for T21 varies between 19 and 44%, and depends on maternal age and gestational age at prenatal testing [22]. Since our data set on ToPs, information of maternal age was partially missing, overall estimates of foetal loss for women of any age were used. The correction factors of natural foetal loss that we used for women screened by AC was 25% and for women screened by CVS 32%. These factors are based on a study of Savva et al. where foetal loss at different maternal ages were estimated by survival analysis using follow-up of 5177 prenatally diagnosed cases [23]. The impact of prenatal screening is defined as the difference between the natural livebirth prevalence and actual livebirth prevalence, divided by the natural livebirth prevalence.

#### Statistical analyses

Linear regression analyses were used to analyse time trends in mean maternal age, the impact of prenatal screening and T21 livebirths prevalence. *Χ*^2^ tests were used to investigate whether the proportions of invasive tests, positive T21 diagnoses and ToP subsequent T21 diagnoses were different between the period before (2000–2006) and after (2007–2013) the implementation of the national screening program. A *p-*value < 0.05 (two sided) was considered to be statistically significant. All statistical analyses were performed using IBM SPSS 20.0.

### Results

All livebirths in the Netherlands recorded by CBS are included in the period 2000–2013 (*N* = 2 641 447). The total number of annual livebirths decreased from 206,619 births in 2000 to 171,426 births in 2013.

#### Maternal age

**Fig. 2:**
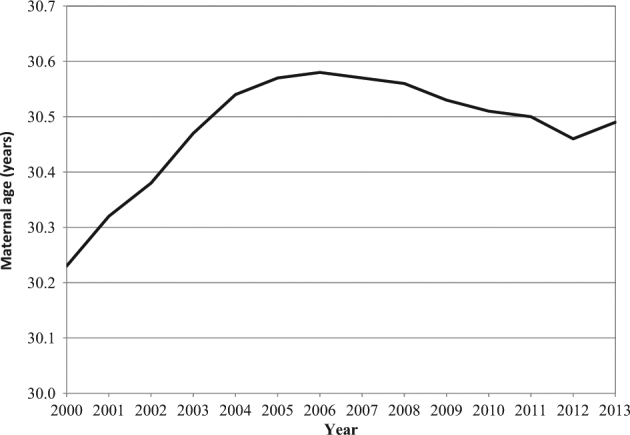
Mean maternal age in the Netherlands, 2000–2013

**Fig. 3:**
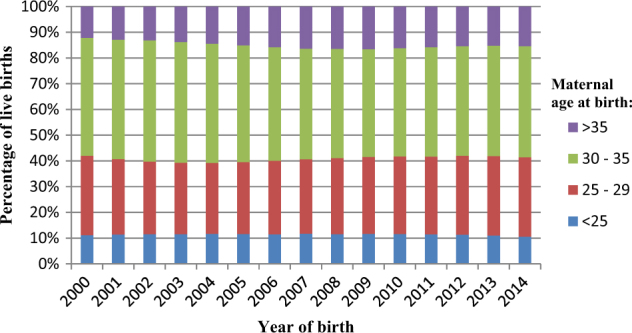
Maternal age distribution in the Netherlands, 2000–2013

The mean maternal age has slightly increased since 2000 from 30.2 years to 30.5 years in 2013 (Fig. 2), leading to a significant increasing trend of 0.014 year (95% CI: 0.001–0.027; *p* = 0.04). Because maternal age is reported as a discrete variable (age last birthday) in the CBS tables, one could add 6 months on top of the mean maternal age from 30.7 years in 2000 to 31.0 years in 2013. The gradual increase of mean maternal age would seem to have stopped in around 2006. The proportion of mothers aged ≥ 36 years has increased from 12.2% in 2000 to 16.6% in 2009, to gradually decrease afterwards to 15.2% in 2013 (Fig. 3).

#### Invasive tests

During the period 2000–2013, a total of 127 077 of invasive tests were performed. Expressed as proportion of the total livebirths, the number of invasive tests has steadily decreased with 0.18% a year (95% CI: −0.21 to −0.15), from 5.9% in 2000 to 3.2% in 2013 (*p* < 0.001).The proportion of invasive tests decreased from 5.4% before to 4.2% after implementation of the national screening program in 2007 (*p* < 0.001). In 2000, advanced maternal age was the main reason for invasive testing (73%), in 2013 this was reduced till 28%. In contrast to the decreasing trend of invasive tests performed, the proportion of positive T21 diagnoses from these tests has increased from 1.6% in 2000 to 4.8% in 2013, with a significant increase of 0.22% a year (95% CI 0.18 to 0.26; *p* < 0.001). The proportion of prenatal diagnosis after invasive tests increased from 2.1% before to 3,6% after 2007 (*p* < 0.001). The proportion of ToP subsequent to T21 diagnosis was on average 85.7%, fluctuating between 79.3% and 93.9%, with no clear time trend (*p* = 0.11). Also no significant change in proportion of ToP was found before and after 2007 (*p* = 0.08). After T21 diagnosis by AC, the pregnancy was terminated less often compared to CVS, with a range 73.6% till 93.6% and 83.6% till 96,4%, respectively (Table 1). The impact of prenatal screening increased with 29.2% in 2000 till 40.8% in 2013 and showed a significant time trend of 0.86% a year (95% CI: 0.32 to 1.40; *p* < 0.001).

**Table 1:** The trend in prenatal testing and reproductive choice after confirmed trisomy 21 (T21) diagnosis in the Netherlands, 2000–2013

Year	Total livebirths	Number of invasive tests	Invasive tests versus total livebirths (%)	Proportion AC of all invasive tests (%)	Proportion CVS of all invasive tests (%)	Number of confirmed T21 diagnosis	T21 diagnosis following invasive test (%)	Number of ToPs after T21 diagnosis	Proportion of ToP after T21 diagnosis by AC (%)	Proportion of ToP after T21 diagnosis by CVS (%)	Proportion of ToP after T21 diagnosis (%)	ToP versus total livebirths (%)
2000	206619	12097	5.9	73.1	26.9	189	1.6	162	82.5	91.3	85.7	0.08
2001	202603	11433	5.6	73.6	26.4	179	1.6	158	85.6	91.5	88.3	0.08
2002	202083	11600	5.7	72.2	27.8	209	1.8	184	84.9	92.8	88.0	0.09
2003	200297	10940	5.5	72.4	27.6	231	2.1	217	93.6	94.3	93.9	0.11
2004	194007	9910	5.1	69.1	30.9	253	2.6	226	83.7	96.4	89.3	0.12
2005	187910	9538	5.1	67.4	32.6	232	2.4	184	69.6	88.9	79.3	0.10
2006	185057	8793	4.8	66.1	33.9	249	2.8	206	76.7	89.2	82.7	0.11
2007	181336	8528	4.7	67.5	32.5	262	3.1	217	76.7	88.0	82.8	0.12
2008	184634	8660	4.7	66.7	33.3	293	3.4	252	78.7	92.8	86.0	0.14
2009	185158	8056	4.4	67.1	32.9	270	3.4	241	85.5	93.2	89.3	0.13
2010	184325	7451	4.0	66.4	33.6	307	4.1	266	80.6	92.8	86.6	0.14
2011	180020	7504	4.2	64.5	35.5	255	3.4	213^a^	81.1	85.7	83.5	0.12
2012	175972	7067	4.0	63.7	36.3	258	3.7	205	73.6	83.6	79.5	0.12
2013	171426	5500	3.2	58.0	42.0	265	4.8	221^a^	77.5	88.3	83.4	0.13

We have estimated this number by assuming that in Nijmegen the percentage ToPs in the prenatal diagnosis was similar to that in the other centres

*AC* amniocentesis, *CVS* chorionic villus sampling, *ToP* termination of pregnancy

^a^ In 2011 and 2013, the WPDT annual reports contained no information on the number of ToPs of the centre in Nijmegen

#### Livebirth prevalence of T21

**Fig. 4:**
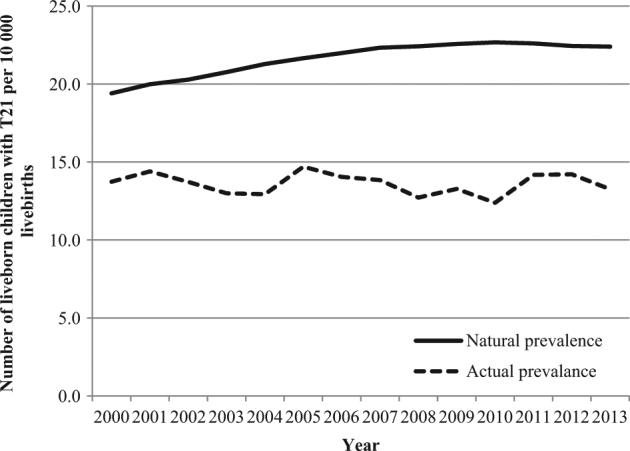
Predicted livebirth prevalence of T21 in the Netherlands, 2000–2013. Natural prevalence: without prenatal diagnosis and termination of pregnancy. Actual prevalence: with prenatal diagnosis and termination of pregnancy

An integration of these data resulted in an estimated livebirth prevalence of T21 that remained quite stable, ranging from 12.4 to 14.7 per 10,000. The mean livebirth prevalence of T21 for the period of 2000–2013 was 13.6 per 10,000 livebirths (Fig. 4). No significant trend in mean livebirth prevalence of T21 over the years 2000–2013 (regression coefficient −0.025 (95% CI: −0.13 to 0.77; *p* = 0.60) was found. A decline of the total number of livebirths resulted in a decrease in the absolute number of livebirths with T21 from 284 to 227 (Table 2).

**Table 2:** Estimated livebirth prevalence of T21 in the Netherlands, 2000–2013

Year	Total livebirths	Expected number of T21 livebirths	Natural livebirth prevalence of T21 per 10 000	AC_ToP	CVS_ToP	Actual number of T21 livebirths	Impact of prenatal screening (%)	Livebirth prevalence of T21 per 10 000
2000	206 619	401	19.4	99	63	284	29.2	13.7
2001	202 603	405	20.0	83	75	292	28.0	14.4
2002	202 083	410	20.3	107	77	277	32.3	13.7
2003	200 297	416	20.8	117	100	260	37.4	13.0
2004	194 007	413	21.3	118	108	251	39.2	12.9
2005	187 910	407	21.7	80	104	276	32.1	14.7
2006	185 057	407	22.0	99	107	260	36.1	14.0
2007	181 336	405	22.3	92	125	251	38.0	13.8
2008	184 634	414	22.4	111	141	235	43.3	12.7
2009	185 158	418	22.6	118	123	246	41.2	13.3
2010	184 325	418	22.7	125	141	228	45.4	12.4
2011	180 020	407	22.6	99	114	255	37.3	14.2
2012	175 972	395	22.4	78	127	250	36.7	14.2
2013	171 426	384	22.4	93	128	227	40.8	13.3

Actual number of T21 livebirths = expected number of T21 livebirths − ((AC_ToP × 0.75) + (CVS_ToP × 0.68))

Impact of screening (%) = (expected number of T21 livebirths − actual number of T21 livebirths) / expected number of T21 livebirths

The correction factor for natural foetal loss for women screened by AC was 25% and CVS was 32%

*T21* trisomy 21, *AC*_*ToP* termination of pregnancy subsequent positive amniocentesis, *CVS*_*ToP* termination of pregnancy subsequent positive chorionic villus sampling

## Discussion

In this study, we found a stable livebirth prevalence of T21 in the Netherlands of 13.6 per 10,000 livebirths from 2000 to 2013. The effect of actively informing all pregnant women about prenatal screening tests, starting around 2004, and resulting in a national screening program in 2007, has not led to a decrease in livebirth prevalence of T21, as estimated by our model.

*Model* The model described in this study was based on maternal age and maternal age-specific T21 risk factors [19, 20]. Using maternal age-specific risk factors to estimate expected T21 pregnancies is a method used internationally, as no large differences have been found when compared to the empirical data. Especially for time trends, no systematic differences are to be expected [24–26]. De Graaf et al. (2011) used a similar model to estimate the livebirth prevalence of T21 in the Netherlands. They validated the results using empirical data of postnatal T21 diagnoses. Birth numbers estimated by the theoretical model were 4% lower, resembling a mean difference of 0.5 per 10 000 births lower, compared with empirical data [6]. Although there was a slight underestimation of birth prevalence, the differences between both methods were small and the time trends in birth prevalence were similar.

### Trends in factors

First, advancing maternal age is a known risk factor for T21. In the Netherlands, maternal age slightly increased till around 2006; however, it did not rise any further. The proportion of mothers aged ≥36 years has increased from 2000 to 2009, but gradually decreased till 2013. Furthermore, a decreasing trend in the number of performed prenatal invasive tests is present. Before 2007 FCT was already being used in pilot studies and women older than 35 years already had direct access to invasive testing. After introduction of the FCT and the structural anomaly scan prenatal diagnostic testing was used more effective, meaning mainly women identified as having a high risk for T21 based on FCT or based on ultrasound anomalies made use of prenatal invasive tests [27, 28]. Because screening tests are more precise than using age criterion a lower proportion of women underwent invasive testing for maternal age criterion alone [10].

The increase in the proportion of positive T21 diagnoses through diagnostic testing from 2000 to 2013 can be explained by a more accurate risk assessment for pregnant women, as only high-risk women are referred for confirmation by invasive testing [28, 29]. Furthermore there is a slight improvement to the test performance of the FCT [30]. The proportion of induced abortions subsequent to T21 diagnosis was on average 85.7%, fluctuating between 79.3 and 93.9%, with no clear time trend. More prenatal diagnoses of T21 in combination with a stable ToP rate after a prenatal diagnosis counterbalanced the increase of the natural livebirth prevalence by increasing maternal age (Fig. 4). In other words, the impact of prenatal testing increased. The decision for ToP is a difficult one to take and an emotional challenge influenced by many personal, social, cultural and psychological factors [16, 31]. In general, the proportion of pregnancy terminations in the Netherlands is the lowest of all Western countries, being around 8.5–9 per 1000 [32, 33]. This can be explained by the fact that norms and values of society with regard to childbirth and termination has considerable influence on the decision to terminate a pregnancy. In the Netherlands, contraception and communication concerning sexual activities and the potential consequences are readily available for young women. Furthermore, the natural character of pregnancy is highly valued; pregnancy and delivery are generally considered as non-medical events that one should not ‘unnecessarily’ interfere with [34].

### Livebirth prevalence of T21

The estimated livebirth prevalence remained stable during 2000–2013. The introduction of the nationwide screening program in the Netherlands did not lead to a sharp decline around 2007 but this can be explained by the fact that FCT was already being used in pilot studies before 2007 and women older than 35 years already had direct access to invasive testing. Another breaking point in livebirth prevalence could have been 2004. Before 2004 physicians were not allowed to actively inform pregnant women about screening, unless there was a preceding medical reason (for instance maternal age or a previous child with a trisomy in the family). Since 2004, this was allowed which already caused an increase in the uptake of prenatal screening tests before 2007. However, no decline of livebirth prevalence was found in 2004. The reason for a stable livebirth prevalence may be the fact that FCT uptake remained relatively low (27%) compared to other European countries. It has been shown that the uptake of prenatal testing is strongly associated with the screening policy of a certain country [4, 35]. Screening uptake in the Netherlands may have been influenced by the additional costs for T21 screening (€160) compared to the standard antenatal care. Furthermore, there is a public debate that precedes the implementation of the T21 screening program, and the ‘right not to know’ and respect for autonomous choice in the Netherlands [35]. A different aspect may be the way midwives are counselling pregnant women by focussing on relationship building and health education, instead of informed decision making [36]. Bakker et al. analysed 820 questionnaires of women in the Netherlands and the main reason for the low uptake of the FCT was the relatively positive attitude towards DS and a negative attitude towards TOP [37].

Reported data about T21 livebirth prevalence over the last decades vary. For 2003, Weijerman et al. (2008) reported a T21 prevalence of 16 per 10,000 livebirths [38], while European Registration of Congenital Anomalies (EUROCAT) concluded that T21 livebirth prevalence was 15 per 10,000 [39]. De Graaf et al. (2011) reported a prevalence of 11 per 10,000 livebirths in the early 1990s, increasing to around 14 per 10,000 in 2007 [6]. Van Gameren-Oosterom et al. (2012) concluded that the proportion of T21 livebirths did not change during the period 1997–2007, with a mean prevalence of 14.5 per 10,000 births, 85% of which were livebirths [40]. This would correspond to 12.3 per 10,000 livebirths. Recently, a cohort study performed in the Northern Netherlands showed a total prevalence of T21 of 23.3 per 10,000 births with 57% livebirths in 2005–2006 and 56% in 2007–2012. This would correspond to 13.2 per 10,000 livebirths [41]. The reason for this slightly lower livebirth prevalence may be the lower maternal age of the northern region of the Netherlands compared to other regions [42]. A very recent study by de Graaf et al. (2017) shows an increase of livebirth prevalence from 11.6 per 10,000 in 1991 to a peak of 15.9 per 10,000 in 2002, followed by a slight decrease to 13.4 per 10,000 in 2013 (and 11.1 per 10,000 in 2015). Livebirth estimates were based on numbers of postnatal T21 diagnoses by cytogenetic centers and non-ToP numbers of prenatal T21 diagnoses in WPDT reports. A correction had to be made for livebirths and natural foetal loss in the non-ToP category. De Graaf et al. modelled this correction in two different ways. In both scenarios, there is a decreasing livebirth prevalence after 2002. Estimates for 2014 and 2015, as regards the percentage of livebirths after a prenatal diagnoses, were based on trend data from preceding years [39]. It is surprising that de Graaf et al. find a decreasing trend also only for 2000 till 2013 and in the current study no such trend was found. How can this difference in trend be explained? First of all, the major limitation of these studies is that the estimates of number of livebirths with T21 are not completely based on actual counts. Present study is based on a model using maternal age in general population, and the data on ToP and natural loss in case of T21. The study of de Graaf et al. models the number of livebirths with T21 in the number of reported non-ToPs. However, the current model of Morris et al. leads to an estimation of livebirth prevalence of on average 13.6 per 10,000 for the period 2000–2013, which is the same as found in the recent study of de Graaf et al. for the same period of time. There was no specific maternal age information, so the overall estimates of natural foetal loss rate were used. However, overestimates of the foetal loss rate in younger mothers will cause the weight of prenatally diagnosed cases to be too low, which will lead to an overestimation of the actual livebirth prevalence. Also, underestimation of the foetal loss rate in older mothers will cause the weight of prenatally diagnosed cases to be too high, which will lead to an underestimation of the expected livebirth prevalence. Finally, it may be possible that we found a stable livebirth prevalence by chance because the 95% confidence intervals in such a model approach as used in the current study on an annual basis are fairly large.

Because NIPT became available in 2013 in neighbouring countries, overestimation of T21 livebirth prevalence is possible. In the Netherlands, NIPT became available only for women at increased risk for T21 after the FCT in April 2014. This led to about 3000–3600 Dutch women crossing the border to undergo NIPT at their own costs [43]. The numbers of T21 cases in this low-risk population is probably too small to affect our estimates, since perhaps almost all T21 diagnoses are confirmed using invasive diagnostic testing in the Netherlands and thereby registered in the database of the WPDT currently used. [44]

In the Netherlands, there is no national registry system for T21. EUROCAT is a regional system of Northern Netherlands and does not cover the whole country. The Perinatal Registry Netherlands foundation (PRN) contains records of all infants born from 16 weeks of gestation under the care of a midwife at home or in a hospital, as well as those born under the care of an obstetrician in a hospital within the first 28 days of life. Similar to EUROCAT, this registration is done on a voluntary basis. In addition, no confirmatory genetic test is needed for the registration of DS. As a result from the voluntary basis, it is likely that underestimation is present. A national registry system that collects complete data of childbirth and T21 diagnosis is needed to ensure baseline data.

## Conclusion and future research

This study showed a stable livebirth prevalence of T21 with a mean of 13.6 per 10,000 in the Netherlands during the period 2000–2013. The national screening program in 2007 for all pregnant women seems to have had limited impact on the livebirth prevalence of T21.

Qualitative studies could provide more insights into whether and how parents make their reproductive decisions. Since April 2017 non-invasive prenatal testing (NIPT) is introduced in the Netherlands as a first-tier screening test as alternative for the FCT. The media has generated intense debate about DS and the NIPT screening test. The question remains if the livebirth prevalence of T21 will change. In our opinion a national registry is needed to ensure baseline data. Currently, the data on the number of postnatal diagnoses can be derived from the cytogenetic centers. However, individual centres have to be approached. The recommendation is to collect these data on annual basis in a national registry. Data on prenatal diagnoses and reported ToPs can be derived from the WPDT reports. However, accurate reporting is required to avoid that multiple versions of WPDT reports are circulating. Furthermore in the non-ToPs category, a distinction could be made in the categories ‘livebirths’, ‘natural loss/still births’, and ‘unknown outcomes’. A national register could contain further maternal and neonatal information as well.
